# The Development of the Municipal Registry of People with Diabetes in Porto Alegre, Brazil

**DOI:** 10.3390/jcm13102783

**Published:** 2024-05-09

**Authors:** Rafael Dal Moro, Lucas Helal, Leonel Almeida, Jorge Osório, Maria Ines Schmidt, Sotero Mengue, Bruce B. Duncan

**Affiliations:** 1 Postgraduate Program in Epidemiology, Universidade Federal do Rio Grande do Sul, Porto Alegre 90035-003, Brazil; 2 Secretaria Municipal de Saúde de Porto Alegre, Porto Alegre 90010-150, Brazil

**Keywords:** diabetes mellitus, registries, routinely collected health data, complications, primary healthcare, hospitalization, mortality

## Abstract

**Background/Objective:** Diabetes registries that enhance surveillance and improve medical care are uncommon in low- and middle-income countries, where most of the diabetes burden lies. We aimed to describe the methodological and technical aspects adopted in the development of a municipal registry of people with diabetes using local and national Brazilian National Health System databases. **Methods:** We obtained data between July 2018 and June 2021 based on eight databases covering primary care, specialty and emergency consultations, medication dispensing, outpatient exam management, hospitalizations, and deaths. We identified diabetes using the International Classification of Disease (ICD), International Classification of Primary Care (ICPC), medications for diabetes, hospital codes for the treatment of diabetes complications, and exams for diabetes management. **Results:** After data processing and database merging using deterministic and probabilistic linkage, we identified 73,185 people with diabetes. Considering that 1.33 million people live in Porto Alegre, the registry captured 5.5% of the population. **Conclusions:** With additional data processing, the registry can reveal information on the treatment and outcomes of people with diabetes who are receiving publicly financed care in Porto Alegre. It will provide metrics for epidemiologic surveillance, such as the incidence, prevalence, rates, and trends of complications and causes of mortality; identify inadequacies; and provide information. It will enable healthcare providers to monitor the quality of care, identify inadequacies, and provide feedback as needed.

## 1. Introduction

Diabetes mellitus is a significant and escalating public health challenge, particularly in low- and middle-income countries (LMICs). Its burden is rapidly rising in most countries [1]. Projections indicate a global increase from 537 million in 2021 to 783 million people living with diabetes in 2045, with 94% of this increase occurring in LMICs [2]. In Brazil, the prevalence of diabetes increased by 24% from 2013 to 2019 [3] and is expected to grow from the sixth to the third leading cause of death by 2040 [4].

Enhancing information and communication technology is a pivotal strategy to face the challenges posed by the diabetes burden [1]. To that end, real-world data [4,5] obtained from health information systems have been harnessed to elucidate health problems and determinants of population health [6,7]. By integrating and pairing multiple health data sources, population registries are often essential in the secondary use of real-world data [8]. They yield new information for a given problem in a specific population by leveraging pre-existing information, in accordance with the Global Digital Health Strategy from the World Health Organization, encouraging the use of secondary health data to enhance the quality of healthcare and research effectiveness [9,10].

Population registries related to diabetes have been developed in high-income countries but are uncommon in LMICs [11,12]. To the best of our knowledge, functional population-based diabetes registries constructed with routinely collected health system data are absent in Latin America. In Brazil, information systems pertaining to primary care are now available to complement those already used for hospitalization and deaths [13,14]. Brazil’s vast geographical expanse and regional specificities have led to municipal initiatives to solve local problems [15,16]. Since 2000, public health authorities in Porto Alegre, in southern Brazil, have implemented various information systems complementary to existing national ones to support clinical, regulatory, and health surveillance actions.

Considering the scarcity of population-based records on diabetes care and complications in LMICs and aiming to further qualify municipal healthcare actions, we developed a municipal registry of people with diabetes in Porto Alegre, Brazil. Here, we document the construction of this registry and discuss its potential uses and the challenges ahead for its integration into epidemiological surveillance and clinical care.

## 2. Materials and Methods

Porto Alegre is the capital of Rio Grande do Sul, the southernmost state in Brazil, having approximately 1.33 million inhabitants in 2022 [17]. Public health provision in Porto Alegre is conducted by the Municipal Health Department (Secretaria Municipal de Saúde [SMS], Porto Alegre, Brazil), which administers local aspects of the Brazilian National Health System (Sistema Único de Saúde, [SUS]) care through about 190 outpatient services, as well as 14 in-house and outsourced hospital services, in total comprising more than 3900 health professionals working in healthcare, patient flow management, and epidemiologic surveillance [18]. 

### 2.1. Data Sources and Ethical Considerations

After the research ethics committees of the SMS and Hospital de Clínicas de Porto Alegre approved the study project, we obtained eight databases from seven clinical and administrative data information systems, which are briefly described below: 1. Medications Dispensing System (Dispensação de Medicamentos—DIS); 2. Primary Care Electronic Health Record (Prontuário Eletrônico da Atenção Primária—e-SUS APS); 3. Consultations and Exams Management (Gerenciamento de Consultas e Exames—GERCON); 4. Exams Management (Gerenciamento de Consultas e Exames—GERCON); 5. Hospitalization Management (Gerenciamento de Internações—GERINT); 6. Hospital Billing (Faturamento Hospitalar, Sistema de Informações Hospitalares—SIH); 7. Hospital Information System (Sistema de Informações Hospitalares—SIHO); and 8. Mortality Information System (Sistema de Informações de Mortalidade—SIM). 

It is noteworthy that the information systems and their respective databases complement each other by encompassing various contexts and diverse points of contact between individuals and healthcare services. These range from routine primary care appointments to other health procedures, as well as the administration of medications in outpatient settings, specialized consultations, and diagnostic tests conducted under specific circumstances. Additionally, they encompass hospitalizations, less frequent emergency interventions, and ultimately, terminal outcomes such as mortality, particularly in cases where previous databases failed to identify diabetes-related events throughout the period covered by the registry of an individual’s lifespan. A brief description of each data system follows. Only the primary author (Dal Moro, R), as an employee of the Municipal Health Department, had direct access to the data and constructed the necessary linkages.

#### 2.1.1. Outpatient Medications (DIS)

We acquired data on outpatient medications from the Medication Dispensing System (DIS), developed in Porto Alegre and employed in public pharmacies and municipal-level public health system dispensaries since 2017. The DIS was designed to streamline the processes involved in the transport, storage, dispensation, and disposal of medications listed on the Municipal List of Medicines (Lista Municipal de Medicamentos—Remume) [19]. The REMUME encompasses the essential medications and resources for diabetes care as defined by Brazilian federal law [20] and the Municipal Program for Distribution for Diabetes Medications and Supplies (Programa Municipal de Distribuição de Insumos para Diabetes, PMDID), a complementary action of pharmaceutical care in Porto Alegre that guarantees access to insulin and supplies for home-based capillary blood glucose monitoring [21].

#### 2.1.2. Primary Care (e-SUS APS)

We gathered primary care encounter data from the Electronic Primary Care Health Record System, developed by the Brazilian Ministry of Health (e-SUS APS) and implemented in Porto Alegre in 2014. This system documents both spontaneous and scheduled encounters provided by primary healthcare facilities. By 2021, the city’s primary care network had 142 health services.

#### 2.1.3. Specialized Care and Outpatient Exams (GERCON Consultation and Examination Management Systems)

Data on specialized care were obtained from the Consultation and Examination Management System, an information system conceived and developed in Porto Alegre and utilized since 2016. This system facilitates requesting, regulating, scheduling, and confirming consultations in the specialized healthcare network. In January 2020, the system was expanded to include an outpatient exam module, incorporating a second database into the registry development process. 

#### 2.1.4. Hospitalizations (GERINT and SIH)

For hospitalization information, we obtained data from two sources. The first was the Hospitalization Management System (GERINT), a state-level integrated system introduced in 2017 to enhance patient flow management and access to hospital beds, financed by the SUS in Rio Grande, including Porto Alegre. The second was the Hospital Billing System (SIH), developed by the Brazilian Ministry of Health to document the billing and financial resource transfer related to hospital admissions.

#### 2.1.5. Urgent Care (SIHO)

Urgent care data were gathered from a local patient health record system (SIHO) used in Porto Alegre since the mid-2000s. This system records urgent and emergency encounters paid for by the SUS.

#### 2.1.6. Mortality (SIM)

We extracted data on deaths from the Brazilian Mortality Information System (SIM), which the Brazilian Ministry of Health has developed to oversee national mortality data since 1975.

### 2.2. Data Linkage, Database Generation, and Data Management

We chose variables from the eight databases mentioned above to facilitate (a) linkage across healthcare datasets to enable the integration of exposures and outcomes, (b) elaboration of a demographic profile of participants, and (c) identification of diabetes and its complications. Subsequently, we extracted the target data from data servers of the SMS and PROCEMPA, the entity that develops and maintains Porto Alegre’s municipal information systems. The databases were stored in a computing environment dedicated to data management. We also implemented data protection procedures for privacy and individual safety, including access control to the data through different privilege levels through a two-factor authentication process. Data usage has been performed strictly for scientific research purposes. We operated the data through an exclusive relational database using PostgreSQL 13.0 in a DBeaver 21.0 integrated development environment for this stage. 

Any of the following criteria identified a possible case of diabetes (Table 1): (a) a code for diabetes according to the International Classification of Diseases—version 10 (ICD-10) or the International Classification of Primary Care—version 2 (ICPC-2); (b) a medication prescribed and dispensed to control diabetes; or (c) a procedure code indicating treatment for acute or chronic complications of diabetes, according to the Brazilian public health code for procedures and health materials (Tabela de Procedimentos, Medicamentos e Órteses-Próteses-Materiais Especiais do Sistema Único de Saúde). For death records, we identified diabetes among both the underlying and the contributing causes.

We identified people with diabetes separately in each database, considering the occurrence of events between July 2018 and June 2021. The earliest occurrence was selected when a person had two or more occurrences in the same database. Thus, we obtained eight intermediate databases containing a unique record to identify each person who met the criteria for diabetes. We joined these eight intermediate databases to create the municipal registry of people with diabetes. 

To compare bases and deduplicates when needed, we used the unique identification number as a deterministic matching key, except for the hospitalization billing database (SIH) and mortality information system (SIM). For these latter bases, we applied the Jaro–Winkler distance algorithm [22] (R Package 4.1.2, 64 bits, for Windows; RStudio 2021.09.1. for Windows; Fedmatch package 2.0.4 for R). For comparison, we used the variable names and the date of birth, accepting a minimum similarity value of 0.95 (on a scale from 0 to 1, where 1 indicates total similarity). When the same person was listed in more than one intermediate database, we registered the presence of diabetes based on the database containing the earliest occurrence.

### 2.3. Statistical Methods

Descriptive statistical analyses were conducted, estimating the absolute and percentage frequencies for categorical variables and the means and standard deviations for numerical variables. The analyses were performed using the R Software Package version 4.1.2 (64-bit) for Windows and RStudio version 2021.09.1 for Windows.

## 3. Results

This version of the municipal registry of people with diabetes in Porto Alegre (July 2018–June 2020) contains the following data for each person: unique identifier number, date of birth, sex, diagnostic code or text (ICD, ICPC-2, medication, or procedure code) and the date diabetes was first registered during the study period in the health system databases.

**Figure 1:**
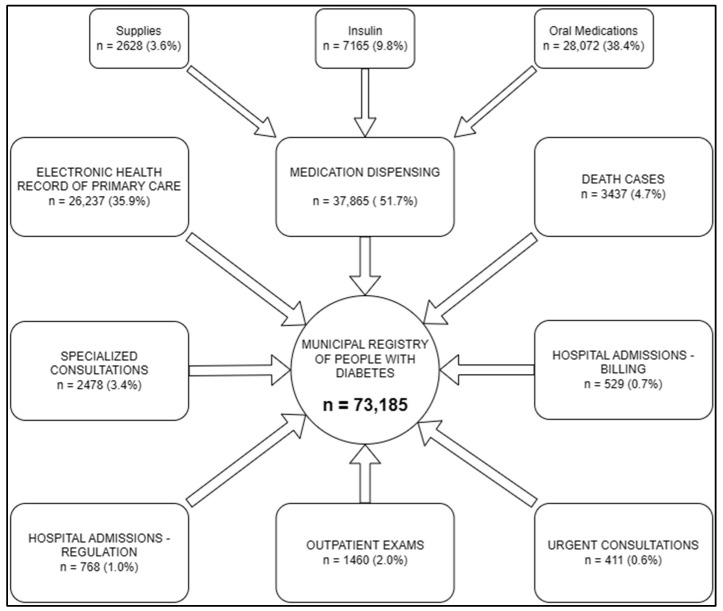
Municipal registry of people with diabetes in Porto Alegre, according to the origin and number of people identified.

This initial data processing identified 1,007,850 occurrences of diabetes from 8 databases. The deduplication process yielded 73,185 unique individuals with diabetes. Of these, 33,050 were observed in 1 database, 24,755 in 2 databases, and 15,380 in 3 or more databases. Regarding the identification sources, 37,865 individuals were identified based on antidiabetic medication dispensed; 26,237 on primary care consultations; 3437 from the mortality registry; 2478 through specialized consultations; 1460 via specific laboratory exams; 1297 from hospital admissions or billing; and 411 from urgent care consultations (Figure 1). Considering the estimated population of Porto Alegre in 2020 to be 1.33 million, this represents 5.5% of the population over the three-year period.

The registry was designed to allow future processing and extraction to obtain additional information regarding preventive actions (e.g., ophthalmological evaluation, diabetic foot assessment, tracking of creatinine and microalbumin), smoking habits, control measures (Hb1Ac, glucose level, blood pressure, lipid profile), treatment profile (use of oral medications, insulin use, nutritional monitoring), complications (hypoglycemia, diabetic retinopathy, diabetic nephropathy, diabetic neuropathy, peripheral arterial disease, amputations, hepatic steatosis/fibrosis/cirrhosis, heart diseases, stroke), and both overall and specific mortality. Table 2 shows the feasibility of gathering this information for future analyses based on the 39 items recommended by the International Consortium on Health Outcome Measurement (ICHOM) proposal for data to be included in population registries for diabetes [23].

Among the 39 proposed items, 31 (79.5%) can be gathered through further data processing and extraction. Among them, 19 data items are currently feasible, 15 are immediately available for use, and 4 (body mass index, fasting glucose, blood pressure, and diabetic nephropathy) require further data processing—data engineering, extraction, or cleaning actions. Partial feasibility is defined for 12 (30.7%) items, since we may identify the occurrence but not the detail or result. Most (*n* = 9) are related to laboratory results, as we currently only have an indication that a test was solicited. The Municipality is integrating with clinical laboratories to receive test results, which will then be available for incorporation into the registry. The other three items with partial feasibility (tobacco use and findings from ophthalmologic and foot examinations) will require improvement in the data information systems to inform beyond when an assessment was performed. 

For the remaining eight, we have no primary data source (schooling, date of diagnosis, alcohol consumption, physical activity, lifestyle management, nutritional advice, perception of well-being, and depression score); their inclusion will depend on expanding current information systems. It is possible, for example, to obtain a diagnosis of depression with further data extraction.

## 4. Discussion

The municipal registry of people with diabetes in Porto Alegre identified 73,185 cases at this first data processing (July 2018 to June 2021), almost all receiving care through the SUS. Most were ascertained through primary care encounters or the medication dispensing process. The register permitted the organization of the available data to assess the burden of diabetes, its clinical management, and its complications.

An initial consideration pertains to the quality of the constructed registry, its comparability (adherence to definitions of diabetes used in registries elsewhere), temporal scope (availability of data throughout the analysis period), and completeness of the information gathered (here, both coverage of those with diabetes and the extent of relevant additional data) [11,24]. 

The identification strategy we used is comparable to those widely used [25,26,27], involving (a) diagnosis of diabetes according to international code standards, (b) use of medications specific (or nearly so) for the treatment of diabetes, and (c) procedures for the treatment of diabetes complications according to national codification systems. This process for identifying cases of diabetes has not yet undergone a formal validation study. However, our use of standardized approaches, considering that we only assessed structured or pre-coded data, should have produced consistent and valid information [24]. Previous studies have shown high consistency in the use of the international classification of diseases to identify cases of diabetes [28]. However, information on the use of medications can bring some degree of inconsistency [29], such as the use of metformin to treat prediabetes or other medical conditions. This fact indicates the relevance of future complementary investigations to assess the consistency and accuracy of the assessment of diabetes through recorded medication codes. Analyzing a sample of the original records against the established clinical gold standard investigated through chart review is a further step we plan to undertake to identify our rate of false positives. A capture–recapture analysis should provide an idea of the frequency of missed cases of diabetes.

The registry’s temporal scope is currently limited to a relatively short period, making it challenging to analyze temporal trends. We are increasing the years covered in the registry moving forward, thus augmenting the follow-up time necessary to mount an inception diabetes cohort, i.e., a cohort in which participants can be identified based on the care of their diabetes and not by the occurrence of diabetes complications.

In terms of completeness, three aspects merit mention. The first is the completeness and representativeness of the identified population. Except for the national mortality information system database, the databases were only generated from SUS-provided health services. We did not have access to data generated from private healthcare. In 2019, an estimated 49% of people living in Porto Alegre relied exclusively on the SUS for their healthcare, corresponding to about 652,000 people [30]. Many who did have private plans had ones with limited coverage and continued to use the SUS for other aspects of care. For example, very few private plans cover the cost of medications, and the SUS provides free essential diabetes medications, including insulin, at clinics and through private pharmacies. 

The second is the completeness of the unique personal identification number in each database. A small percentage (5.4%) of records—those of the hospital billings and mortality databases—lacked this identifier, and information about the corresponding events was incorporated into the registry via probabilistic matching. We consider this an acceptable percentage for this first version of the registry. In the foreseeable future, these two national databases will likely switch to include the national unique identification number as a strategy for univocal identification of all public and private health service users [31,32,33]. 

The third aspect of completeness is related to data captured to characterize people with diabetes. Following the International Consortium on Health Outcome Measurement (ICHOM) proposal for data to be included in population registries for diabetes [23], as shown in Table 2, the feasibility of using this registry for future analyses is good, as it permits access to 79.5% of the 39 proposed items. 

**Figure 2:**
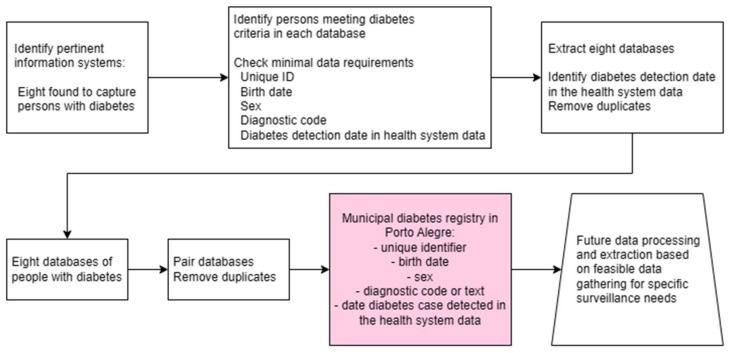
Steps used in the construction of a diabetes registry in Porto Alegre, Brazil (2019–2021), with the perspective of building feasible additions for specific needs of diabetes surveillance and healthcare evaluation.

Our experience provides important contributions to those seeking to undertake similar health system surveillance registries. From an operational perspective, our registry would not be possible without the development of information systems covering the broad range of activities of the SUS in Porto Alegre. Additionally, our access to a unique identifier in the databases simplified and accelerated the process of pairing and the construction of the registry, reducing the computational burden and effort associated with harmonizing the identification data, as already demonstrated in other population registries of diabetes [34,35]. From a strategic point of view, this report demonstrates the feasibility of using real-world secondary data to build new sets of information on the health status of people and populations in a geographically and temporally delimited middle-income country context. A discontinued initiative to create a Brazilian national hypertension and diabetes registry from primary data rather than secondary data like ours likely failed mainly due to the additional burden on primary care personnel of double data entry [36], which is not a problem in our approach. Furthermore, the creation of the registry was only possible through a collaborative effort involving the SUS’s local managers and a local university’s postgraduate program. This combination produced a critical mass of personnel sufficiently well-versed in diabetes, epidemiology, the workings of the SUS, and information technology. Figure 2 outlines the main steps initially taken to build this registry and the future data processing and extraction contemplated for specific needs and interests, notably involving items for which data gathering is entirely feasible.

Considering the continuous qualification and increased data availability, a range of potential new uses can be outlined for the Porto Alegre registry. In the short term, it will be possible to build time series and measure the incidence and prevalence of diabetes, risk factors, acute and chronic complications, and mortality [37,38,39]. In the medium term, monitoring the quality of care and adherence to treatment protocols will be possible as soon as laboratory test results are available. These results will complement opportunities for quality monitoring based on the already available datasets on medications and the use of health services [40,41,42]. Identifying situations of insufficient or inadequate care and alerting professionals and users through digital solutions will also be possible [43,44,45]. In the medium to long term, it will be possible to pair the diabetes registry data with geospatial and socioeconomic data to identify areas of the city of greater epidemiological relevance for strengthening local health services and actions [46,47]. The information produced makes it possible to immediately characterize the population identified with diabetes and thus provide tools for surveillance and epidemiological monitoring of chronic non-communicable diseases at the local level. Finally, the registry´s construction also opens up possibilities for elaborating additional linked studies on various other diseases and conditions of interest to public health. 

It is important to note possible limitations. Many of these are inherent to observational and, especially, secondary data. As already pointed out, the quality of the information produced directly depends on the consistency and completeness of the primary data. Even using structured data, the variability in classification criteria among thousands of health professionals is a limiting factor that generates imprecision in the information. Another limiting aspect is the restriction of the data obtained, which, except for mortality, only comes from the public health system. Although the Brazilian health system provides universal coverage, it does not cover the more expensive medications except in specific cases (e.g., SGLPT2i), and procedures (e.g., elective surgeries), frequently have long waiting lists for which may lead patients to pay for medications and procedures performed in the private sector. Data on the use of these medications and the existence of these procedures are not captured by our databases. These restrictions may prevent a direct extrapolation to the entire local population. As mentioned above, future capture–recapture analyses can estimate the size of the deficit caused by this and other reasons and permit extrapolation to provide estimates of the diabetes prevalence, incidence, and rates of complications. Since diabetes is a disease with slow progression and long duration, the registry s 3-year span currently limits the investigation of the incidence of outcomes. This limitation, however, will diminish over time. Finally, the sustainability of the registry will require further future input of resources. In this regard, a combined effort of the Porto Alegre SMS and the University Federal do Rio Grande do Sul is in motion to create a sound and sustainable foundation for future work.

## 5. Conclusions

The Porto Alegre registry of people with diabetes uniquely identified a relevant fraction of those who had care or events related to diabetes in the SUS in Porto Alegre. With the matched data across the diverse databases it provides, multiple research and epidemiological surveillance questions can be answered, notably those related to deaths and hospitalizations, which can provide information on diabetes complications. This experience can constitute a model for constructing data repositories capable of answering other questions regarding the problems and scenarios of health systems and services in Brazil and other low- and middle-income countries.

## Figures and Tables

**Table 1:** Information systems, databases, main variables, and criteria applied to identify people with diabetes.

Information Systems	Databases	Main Variables	Criteria for Identifying People with Diabetes
Medication Dispensing System	Prescribed and dispensed medications	Medication, prescription date, dispensing date, dispensing location, dispensed quantity	Dispensing metformin, glibenclamide, gliclazide, insulin, or supplies—syringes and needles for insulin application
Electronic Health Record System of Primary Care	Primary care consultations	Date of service, place of service, specialty	ICD (E10 to E14.9) or ICPC-2 (T89 and T90) codes
Consultation and Exams Management System	Specialized consultations	Specialty, diagnostic code, appointment date, referral, and place of care.	ICD codes (E10 to E14.9)
	Outpatient exams *	Requesting unit, request date, type of exam.	ICD codes (E10 to E14.9)
Hospitalization Management System	Hospital admissions—patient flow management system occurrences	Request date, admission date, the diagnostic reason for the request, discharge date, type of bed, and reason for discharge.	ICD codes (E10 to E14.9) or SIGTAP codes for diabetes complication treatment procedures
Hospital Billing System	Hospital admissions—billing occurrences	Date of admission, diagnostic code, procedure performed	ICD codes (E10 to E14.9) or SIGTAP codes for diabetes complication treatment procedures
Hospital Information System	Emergency consultations	Date of care, diagnostic code, procedures, type of discharge	ICD codes (E10 to E14.9)
Mortality Information System	Deaths	Date of death, place of death, main and contributory causes of death	ICD codes (E10 to E14.9)

* Data available only since January 2020.

**Table 2:** Current feasibility and availability of items in the diabetes population registry of Porto Alegre recommended for inclusion by the modified International Consortium on Health Outcome Measurement.

Category	Data Item	Feasibility	Database	Availability
Demographic	Sex	Yes	Multiple	Immediately
	Date of Birth	Yes	Multiple	Immediately
	Race/Ethnicity	Yes	Multiple	Immediately
	Educational Level	No	-	-
Diagnostics Profile	Date of Diagnostics	No	-	-
	Ophthalmologic Evaluation	Partial (Only Occurrence Data)	Specialized Consultations	Immediately
	Diabetic Foot Evaluation	Partial (Only Occurrence Data)	Primary Care Consultations	Needs Data Processing
	Creatinine	Partial (Only Occurrence Data)	Outpatient Exams	Immediately
	Microalbumin	Partial (Only Occurrence Data)	Outpatient Exams	Immediately
	GAD, IA-2, and ICA Antibodies	Partial (Only Occurrence Data)	Outpatient Exams	Immediately
	Body Mass Index	Yes	Primary Care Consultations	Needs Data Processing
Lifestyle	Tobacco Use	Partial (ICD Code)	Primary Care Consultations	Needs Data Processing
	Alcohol Consumption	No	-	-
	Physical Activity	No	-	-
Control Measures	HbA1c at Diagnosis Moment	Partial (Only Occurrence Data)	Outpatient Exams	Immediately
	HbA1c Monitoring	Partial (Only Occurrence Data)	Outpatient Exams	Immediately
	Glucose Level at Diagnosis Moment	Partial (Only Occurrence Data)	Outpatient Exams	Immediately
	Glucose Level Monitoring	Partial (Only Occurrence Data)	Outpatient Exams	Immediately
	Blood Pressure	Yes	Primary Care Consultations	Needs Data Processing
	Lipid Profile	Partial (Only Occurrence Data)	Outpatient Exams	Immediately
	Hepatic Enzymes	Partial (Only Occurrence Data)	Outpatient Exams	Immediately
Treatment Profile	Lifestyle Management	No	-	
	Nutritional Management	No	-	
	Oral Medications	Yes	Dispensed Medications	Immediately
	Insulin Treatment	Yes	Dispensed Medications	Immediately
	Insulin Treatment Method	Yes	Dispensed Medications	Immediately
	Fasting Glucose	Yes	Primary Care Consultations	Needs Data Processing
	Other Medications	Yes	Dispensed Medications	Immediately
Acute Events	Severe Hypoglycemia	Yes	Hospital Admissions/Urgent Consultations	Immediately
Chronic Complications	Diabetic Retinopathy	Yes	Specialized Consultations	Immediately
	Diabetic Nephropathy	Yes	Specialized Consultations/High Complexity Procedures	Needs Data Processing
	Diabetic Neuropathic	Yes	Specialized Consultations	Immediately
	Peripheral Arterial Disease	Yes	Specialized Consultations	Immediately
	Amputations	Yes	Hospital Admissions	Immediately
	Heart Diseases	Yes	Specialized Consultations	Immediately
Mortality	All Causes	Yes	Brazilian Mortality Information System	Immediately
	Related to Cardiovascular Diseases	Yes	Brazilian Mortality Information System	Immediately
Patient- Reported Outcomes	Psychological Well-Being	No	-	-
	Depression	No	-	-

## Data Availability

Data are available from the corresponding author upon reasonable request.

## References

[1] GBD 2021 Diseases and Injuries Collaborators (2023). Global, regional, and national burden of diabetes from 1990 to 2021, with projections of prevalence to 2050: A systematic analysis for the Global Burden of Disease Study 2021. Lancet.

[2] International Diabetes Federation (2021). Diabetes Atlas.

[3] Reis R.C.P.D., Duncan B.B., Malta D.C., Iser B.P.M., Schmidt M.I. (2022). Evolution of diabetes in Brazil: Prevalence data from the 2013 and 2019 Brazilian National Health Survey. Cad. Saúde Pública.

[4] Duncan B.B., Cousin E., Naghavi M., Afshin A., França E.B., Passos V.M.d.A., Malta D., Nascimento B.R., Schmidt M.I. (2020). The burden of diabetes and hyperglycemia in Brazil: A global burden of disease study 2017. Popul. Health Metr..

[5] Verschuuren M., van Oers H. (2020). Population health monitoring: An essential public health field in motion. Bundesgesundheitsblatt Gesundheitsforschung Gesundheitsschutz..

[6] Aliabadi A., Sheikhtaheri A., Ansari H. (2020). Electronic health record–based disease surveillance systems: A systematic literature review on challenges and solutions. J. Am. Med. Inform. Assoc..

[7] Kohsaka S., Morita N., Okami S., Kidani Y., Yajima T. (2021). Current trends in diabetes mellitus database research in Japan. Diabetes Obes. Metab..

[8] Nyberg F., Franzén S., Lindh M., Vanfleteren L., Hammar N., Wettermark B., Sundström J., Santosa A., Björck S., Gisslén M. (2021). Swedish COVID-19 Investigation for Future Insights—A Population Epidemiology Approach Using Register Linkage (SCIFI-PEARL). Clin. Epidemiol..

[9] World Health Organization (2022). Digital Adaptation Kit: Family Planning: Operational Requirements for Implementing WHO Recommendations in Digital Systems.

[10] World Health Organization (2021). Global Strategy on Digital Health 2020–2025.

[11] Bak J.C.G., Serné E.H., Kramer M.H.H., Nieuwdorp M., Verheugt C.L. (2021). National diabetes registries: Do they make a difference?. Acta Diabetol..

[12] Naemi R., Shahmoradi L., Islam M.d.S. (2020). Diabetes: From Research to Clinical Practice. Global Experience of Diabetes Registries: A Systematic Review.

[13] Coelho Neto G.C., Chioro A. (2021). A final, quantos Sistemas de Informação em Saúde de base nacional existem no Brasil?. Cad. Saúde Pública.

[14] Barreto M.L., Ichihara M.Y., Pescarini J.M., Ali M.S., Borges G.L., Fiaccone R.L., Ribeiro-Silva R.d.C., Teles C.A., Almeida D., Sena S. (2021). Cohort profile: The 100 Million Brazilian Cohort. Int. J. Epidemiol..

[15] Eugênio Vilaça Mendes (2011). Inovação nos Sistemas Logísticos: Resultados do Laboratório de Inovação Sobre Redes Integradas de Atenção à Saúde Baseadas na APS.

[16] Helena H., Corral A., Ferreira D.P., Troccoli F.T. (2015). Desenvolvimento e Implantação do Módulo de Prontuário Eletrônico do Paciente na SMS de São Paulo. Sec Munic Saúde SP. https://pesquisa.bvsalud.org/portal/resource/pt/sms-9277.

[17] (2023). Censo Demográfico 2022.

[18] Prefeitura Municipal de Porto Alegre, Secretaria Municipal de Saúde Relatório 2o Quadrimestre 2021. http://lproweb.procempa.com.br/pmpa/prefpoa/sms/usu_doc/rg_2_quadrimestre_2021.pdf.

[19] Prefeitura Municipal de Porto Alegre (2020). Secretaria de Saúde. Relação Municipal de Medicamentos Essenciais—REMUME. http://www2.portoalegre.rs.gov.br/sms/default.php?p_secao=960.

[20] Ministério da Saúde Portaria GM/MS No 1.555/2013, de 30 de Julho de 2013. https://bvsms.saude.gov.br/bvs/saudelegis/gm/2013/prt1555_30_07_2013.html.

[21] Prefeitura Municipal de Porto Alegre (2020). Secretaria de Saúde. Programa Municipal de Distribuição dos Insumos para Diabetes. https://www2.portoalegre.rs.gov.br/sms/default.php?p_secao=1091.

[22] Jaro M.A. (1989). Advances in record linkage methodology as applied to matching the 1985 census of Tampa, Florida. JASA.

[23] The International Consortium on Health Outcome Measurement (ICHOM) (2019). Type 1 and Type 2 Diabetes in Adults—Data Collection Reference Guide. https://ichom.org/files/medical-conditions/diabetes-in-adults/dia-reference-guide.pdf.

[24] Chan K.S., Fowles J.B., Weiner J.P. (2010). Review: Electronic Health Records and the Reliability and Validity of Quality Measures: A Review of the Literature. Med. Care Res. Rev..

[25] Venermo M., Manderbacka K., Ikonen T., Keskimäki I., Winell K., Sund R. (2013). Amputations and socioeconomic position among persons with diabetes mellitus, a population-based register study. BMJ Open.

[26] Arffman M., Pirjo I.P., Keskimäki I., Kurkela O., Lindström J., Sund R., Winell K. FinDM Database on Diabetes in Finland. https://urn.fi/URN:ISBN:978-952-343-492-9.

[27] Holman N., Knighton P., Wild S.H., Sattar N., Dew C., Gregg E.W., Khunti K., Valabhji J., Young B. (2021). Cohort profile: National Diabetes Audit for England and Wales. Diabet. Med..

[28] Tang P.C., Ralston M., Arrigotti M.F., Qureshi L., Graham J. (2007). Comparison of Methodologies for Calculating Quality Measures Based on Administrative Data versus Clinical Data from an Electronic Health Record System: Implications for Performance Measures. J. Am. Med Inform. Assoc..

[29] Varkey P., Cunningham J., Bisping S. (2007). Improving Medication Reconciliation in the Outpatient Setting. Jt. Comm. J. Qual. Patient Saf..

[30] (2019). Pesquisa Nacional por Amostra de Domicílios: PNAD 2019.

[31] da Cunha R.E. (2002). Cartão Nacional de Saúde: Os desafios da concepção e implantação de um sistema nacional de captura de informações de atendimento em saúde. Ciência Saúde Coletiva.

[32] Atty A.T.M., Tomazelli J.G., Dias M.B.K., Ribeiro C.M., Migowski A., Bertoni N. (2019). Cartão Nacional de Saúde: Avaliação da Confiabilidade em Bases de Dados Codificadas da Oncologia. Rev. Bras. Cancerol..

[33] Brasil Ministério da Saúde Portaria GM/MS No 2.236, de 2 de Setembro de 2021. GM/MS No 2.236. https://www.in.gov.br/en/web/dou/-/portaria-gm/ms-n-2.236-de-2-de-setembro-de-2021-345783870.

[34] Green A., Sortsø C., Jensen P.B., Emneus M. (2014). Validation of the Danish National Diabetes Register. Clin. Epidemiol..

[35] Akkanen M.J., Kivelä S.L., Koistinen V., Sintonen H., Tuomilehto J. (2009). Inpatient care of patients with type 1 diabetes mellitus by duration of diabetes and sex: A nationwide population-based longitudinal study. Risk Manag. Healthc. Policy.

[36] dos Correia Lo S., Padilha B.M., Vasconcelos S.M.L. (2014). Completitude dos dados de cadastro de portadores de hipertensão arterial e diabetes mellitus registrados no Sistema Hiperdia em um estado do Nordeste do Brasil. Ciência Saúde Coletiva.

[37] National Diabetes Audit, 2015–2016 Report 2a: Complications and Mortality (Complications of Diabetes). NHS Digital; 2017. https://files.digital.nhs.uk/pdf/4/t/national_diabetes_audit__2015-16__report_2a.pdf.

[38] Al-Rubeaan K., Youssef A.M., Ibrahim H.M., Al-Sharqawi A.H., AlQumaidi H., AlNaqeb D., Aburisheh K.H. (2016). All-cause mortality and its risk factors among type 1 and type 2 diabetes mellitus in a country facing diabetes epidemic. Diabetes Res. Clin. Pract..

[39] Rawshani A., Landin-Olsson M., Svensson A.-M., Nyström L., Arnqvist H.J., Bolinder J., Gudbjörnsdottir S. (2014). The incidence of diabetes among 0-34 year olds in Sweden: New data and better methods. Diabetologia.

[40] Ng I.H.Y., Cheung K.K.T., Yau T.T.L., Chow E., Ozaki R., Chan J.C.N. (2018). Evolution of Diabetes Care in Hong Kong: From the Hong Kong Diabetes Register to JADE-PEARL Program to RAMP and PEP Program. Endocrinol. Metab..

[41] Gudbjörnsdottir S., Cederholm J., Nilsson P.M., Eliasson B., Steering Committee of the Swedish National Diabetes Register (2003). The National Diabetes Register in Sweden: An implementation of the St. Vincent Declaration for Quality Improvement in Diabetes Care. Diabetes Care.

[42] Wright C.E., Yeung S., Knowles H., Woodhouse A., Barron E., Evans S. (2018). Factors influencing variation in participation in the National Diabetes Audit and the impact on the Quality and Outcomes Framework indicators of diabetes care management. BMJ Open Diabetes Res. Care.

[43] O’Connor P.J., Sperl-Hillen J.M., Rush W.A., Johnson P.E., Amundson G.H., Asche S.E., Ekstrom H.L., Gilmer T.P. (2011). Impact of electronic health record clinical decision support on diabetes care: A randomized trial. Ann. Fam. Med..

[44] O’Connor P.J., Sperl-Hillen J.M. (2019). Current Status and Future Directions for Electronic Point-of-Care Clinical Decision Support to Improve Diabetes Management in Primary Care. Diabetes Technol. Ther..

[45] Chan J.C.N., Thewjitcharoen Y., Nguyen T.K., Tan A., Chia Y.-C., Hwu C.-M., Jian D., Himathongkam T., Wong K.-L., Choi Y.-M. (2022). Effect of a Web-Based Management Guide on Risk Factors in Patients with Type 2 Diabetes and Diabetic Kidney Disease. JAMA Netw. Open.

[46] Bagheri N., Konings P., Wangdi K., Parkinson A., Mazumdar S., Sturgiss E., Lal A., Douglas K., Glasgow N. (2020). Identifying hotspots of type 2 diabetes risk using general practice data and geospatial analysis: An approach to inform policy and practice. Aust. J. Prim. Health.

[47] Hurst J.E., Barn R., Gibson L., Innes H., Bus S.A., Kennon B., Wylie D., Woodburn J. (2020). Geospatial mapping and data linkage uncovers variability in outcomes of foot disease according to multiple deprivation: A population cohort study of people with diabetes. Diabetologia.

